# Outcomes of Late Corticosteroid Withdrawal after Renal Transplantation in Patients Exposed to Tacrolimus and/or Mycophenolate Mofetil: Meta-Analysis of Randomized Controlled Trials

**Published:** 2011-11-01

**Authors:** A. K. Ali, J. Guo, H. Ahn, J. Shuster

**Affiliations:** 1 *Department of Pharmaceutical Outcomes & Policy, College of Pharmacy, *; 2 *Department of Adult & Elderly, College of Nursing, *; 3 *Department of Health Outcomes & Policy, College of Medicine, University of Florida, USA*

**Keywords:** Steroid withdrawal, Kidney transplantation, Tacrolimus, Mycophenolate mofetil

## Abstract

Background: Corticosteroids are increasingly used in renal transplant patients to minimize organ rejection after transplantation. In attempts to reduce corticosteroids adverse effects, transplant professionals are customary attempted to taper off, and permanently stop corticosteroids after few months of administration with other immunosuppressants.

Objective: To evaluate clinical benefits and risks of late corticosteroid withdrawal in renal transplant patients treated with tacrolimus (TAC) or mycophenolate mofetil (MMF), or both.

Methods: A meta-analysis was performed of published randomized controlled trials that reported outcomes in kidney transplant patients who were randomized to corticosteroids maintenance or late withdrawal under concomitant immunosuppression by TAC, MMF or both. Outcomes included acute graft rejection; graft failure rate; all-cause mortality; incidence of post-transplant diabetes; change in serum creatinine and total cholesterol; and change in pediatric standardized height z-score. PubMed and Google Scholar were used in literature search between 1999 and April 1, 2010. Data were combined using unweighted random effects model.

Results: Nine studies randomized 1907 patients met the inclusion criteria: TAC (n=1); MMF (n=6); both (n=2). Compared to maintenance therapy, late corticosteroid withdrawal was associated with 34% increase in the risk of acute graft rejection (95% CI for OR: 0.47–3.82); 35% and 5% reductions in the risk of graft failure and patient’s all-cause mortality (95% CI for OR: 0.26–1.60; 0.23–3.93, respectively); and 4% increase in post-transplant diabetes risk (95% CI for OR: 0.45–2.41). Late corticosteroid withdrawal was associated with substantial reduction in total cholesterol levels (mean difference: 18.1 mg/dL; 95% CI: 7.1–29.0 mg/dL), but did not reduce serum creatinine levels (0.00 mg/dL; 95% CI: 0.17 to 0.17). Stopping corticosteroids was associated with better pediatric growth outcomes.

Conclusion: Late corticosteroid withdrawal under TAC and/or MMF-lead immunosuppression after kidney transplantation could provide benefits in terms of total cholesterol, patient and graft survival, and pediatric growth. This strategy, however did not reduce the risk of acute graft rejection, post-transplant diabetes mellitus, and deterioration in serum creatinine levels.

## INTRODUCTION

Organ rejection is one of the serious complications of kidney transplantation. Corticosteroids are increasingly used to reduce organ rejection after transplantation. However, corticosteroids have many adverse effects including—but not limited to—hypertension, hypercholesterolemia, glucose intolerance, growth retardation in children, greater incidence of infections, and bone weakness. In attempts to reduce the incidence of graft rejection rates as well as the adverse effects of corticosteroids, transplant professionals are customary attempted to taper off, and permanently stop corticosteroids after few months of administration. Unfortunately, the randomized controlled trials (RCTs) conducted in the 1990s showed increasing rates of acute graft rejection [1, 2]. Cyclosporine and azathioprine were also used as concomitant immunosuppressants for many years. In recent years, both cyclosporine and azathioprine were replaced by more potent agents, tacrolimus (TAC) and mycophenolate mofetil (MMF). Kidney transplant clinicians could taper off corticosteroid under concomitant immunosuppression of either TAC or MMF.

In this study, we performed a meta-analysis of RCTs to evaluate clinical benefits and risks of late corticosteroid withdrawal in kidney transplant patients with concomitant immunosuppression by TAC, MMF, or both.

## METHODS

Data sources and searches

A comprehensive *PubMed* and *Google Scholar* search was independently performed by the authors to find human studies published between 1999 and April 1, 2010 using the search terms kidney transplant, renal transplant, randomized clinical trial, tacrolimus, mycophenolate mofetil, as well as combinations of these terms. The bibliographies of the retrieved literature were also searched for other relevant studies.

Study selection

Clinical trials that met the following criteria were included: randomized controlled trials in kidney transplant patients regardless of age; kidney transplant patients on corticosteroids with concomitant immunosuppression by either TAC or MMF, or both. Late corticosteroid withdrawal in one comparison group was required, which was defined as withdrawing corticosteroids at least three months after transplantation; and the other comparison group was defined as kidney transplant patients who received maintenance corticosteroid therapy with either TAC or MMF, or both. Using either of cyclosporine or azathioprine as concomitant immunosuppressants in addition to the above agents is considered acceptable in the study inclusion criteria. Clinical trials that enrolled organ transplant patients other than kidney, early corticosteroid withdrawal (withdrawal before three months after transplantation), or if the concomitant immunosuppression was by cyclosporine and azathioprine in addition to other agents were excluded.

Data extraction 

Standardized forms are used to extract data from selected studies for patient demographics; inclusion and exclusion criteria; treatment regimens; duration of follow-up (time of randomization post-transplantation; time of data collection post-randomization); total number of patients enrolled; allograft source and characteristics; and clinical outcomes.

Clinical outcomes 

**Figure 1:**
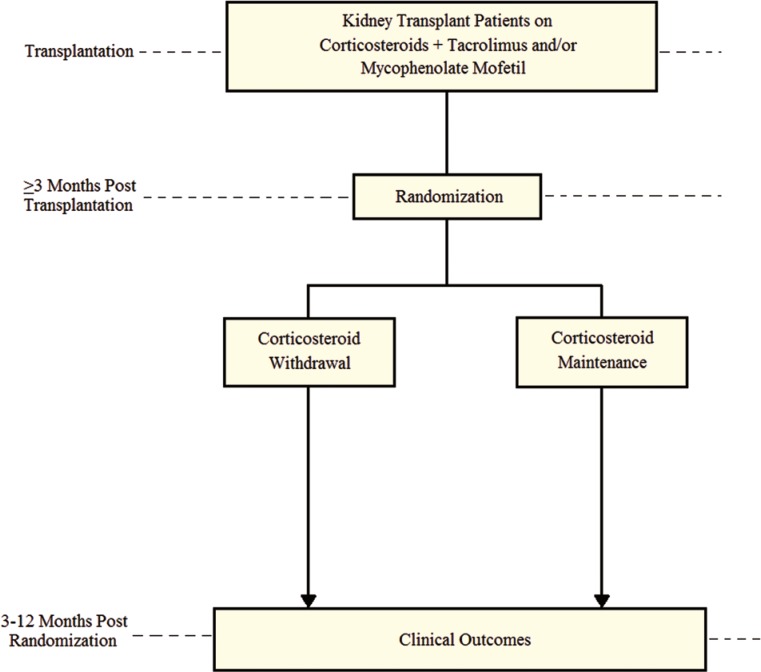
Schematic representation of the study profile for selected trials

Clinical outcomes of interest included the incidence of acute graft rejection; graft failure rate; all-cause mortality; incidence of post-transplant diabetes mellitus; change in serum creatinine and total cholesterol; and change in pediatric standardized height z-score. The definitions of clinical end-points were similar across the trials; three studies reported serum creatinine values in µM/L [3-5], and two studies reported total cholesterol values in mM/L [4, 5]. Both measurements are converted to mg/dL units by dividing serum creatinine values by 88.4 and multiplying total cholesterol values by 28.7. All clinical outcomes were extracted at 3–12 months after randomization (the time of divergence to corticosteroid withdrawal and maintenance) (Figure 1).

Data synthesis and analysis 

Summary odds ratio (OR) and mean difference values with their corresponding 95% confidence intervals (CI) were calculated using unweighted random effects model to combine results from selected studies. A priori 5% level of significance for Type-I error (α) was specified to determine statistical significance. All statistical analyses were conducted using SAS software, ver 9.2 of the SAS System for Windows^®^ (2010 SAS Institute Inc, Cary, NC).

## RESULTS

Overview of trials

**Figure 2:**
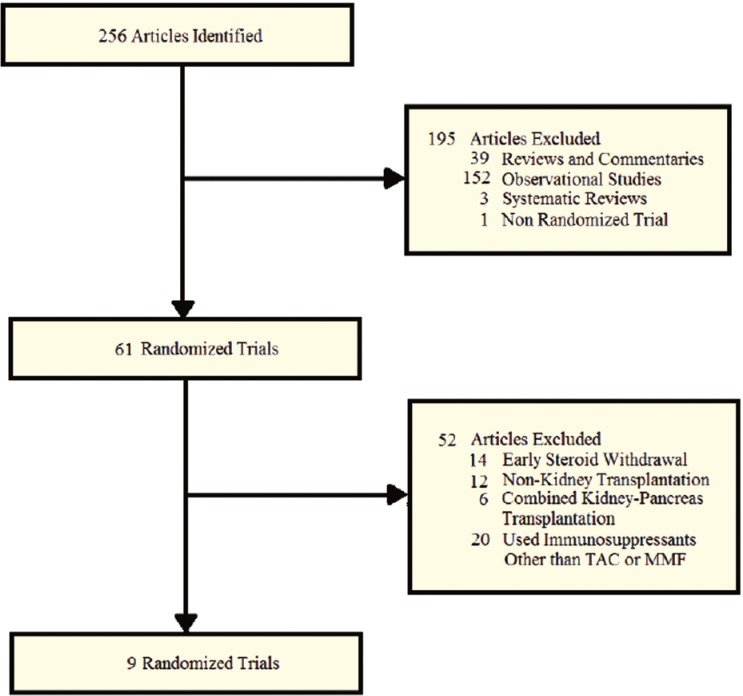
Schematic representation of the review process for study inclusion

A total of 256 (109 from *PubMed*; 136 from *Google Scholar*; 11 secondary bibliographies) potentially eligible studies were identified, and 247 were excluded for not meeting the selection criteria. Nine RCTs were identified for inclusion (Figure 2) [3-11]. One-thousand nine-hundred and seven patients were randomized to late withdrawal or maintenance corticosteroid therapy after kidney transplantation. Six studies included both MMF and cyclosporine [3, 5, 7, 8, 10, 11]; two trials included both TAC and MMF [4, 9]; and one clinical trial included TAC with sirolimus as concomitant immunosuppression therapy [6]. One study did not specify the type of corticosteroid used [3]; three studies used prednisone [5, 8, 11]; one study used methylprednisolone [10]; three trials used both methylprednisolone and prednisone [6, 7, 9]; and one trial used methylprednisolone or equivalent corticosteroid [4]. The mean time of randomization after transplantation was seven (range: 6–15) months. Table 1 shows the characteristics of trials included in the analysis.

**Table 1:** Characteristics of trials included in the meta-analysis

**Trial (Yr)**	**No. of Patients Randomized**	**Follow-Up Time (Months)**		**Treatment Regimen**
		**Post-Transplantation**	**Post-Randomization**	
Benfield, *et al* (2010)	132	6	6	CS**+TAC+SIR
Hocker, *et al* (2010)	42	12	9	CS**+CSA+MMF
Del Castillo, *et al* (2006)	146	6	6	CS+CSA+MMF
Pelletier, *et al* (2006)	118	15	6-12	CS^#^+CSA+MMF
Vanrenterghem, *et al* (2005)	833	3	3	CS*+TAC+MMF
Smak Gregoor, *et al* (2002)	212	6	3-6	CS^#^+CSA+MMF
Sola, *et al* (2002)	92	3	24	CS**+TAC+MMF
Boletis, *et al* (2001)	66	3	3	CS^‡^+CSA+MMF
Ahsan, *et al* (1999)	266	3	9	CS^#^+CSA+MMF
Total	1907			

CSA: Cyclosporine

SIR: Sirolimus

* Methylprednisolone or equivalent

# Prednisone

** Methylprednisolone and prednisone

‡ Methylprednisolone

Table 2 describes characteristics of patients enrolled in the selected trials. Two studies did not report patient age and gender [3,9]; and four studies did not report patient race [3, 5, 9, 10]. The mean age of enrollees was 36.7 (range: 11.6–51.6) years. About 66% (n=1100) of patients were male, and 34% (n=569) were female. The majority of patients were non-African Americans. One clinical trial did not mention the source of the allograft [3]. The majority (77%) of the grafts were from cadaveric sources (n=1354), and about 22% were from live donors (n=403). For most of the patients, the received kidney was their first transplant. Four studies reported donor-recipient mismatch information, with a well-matched donor-recipient characteristics [4, 5, 8, 10].

**Table 2:** Characteristics of patients included in the selected trials

**Trial (Year)**	**Mean Age (Yrs)**	**Gender** **n (%)**		**Race** **n (%)**		**Allograft Source** **n (%)**		**Allograft Characteristics**	
		**Female**	**Male**	**Black**	**White**	**Live Donor**	**Deceased Donor**	**First Transplant ** **n (%)**	**HLA Mismatch** **mean (SD)**
Benfield, *et al* (2010)	11.6	54 (41)	78 (59)	20 (15.2)	99 (75)	88 (66.7)	40 (30.3)	N/A	N/A
Hocker, *et al* (2010)	12.7	14 (33.3)	28 (66.7)	N/A	41 (97.6)	11 (26.2)	31 (73.8)	N/A	N/A
Del Castillo, *et al* (2006)	N/A	N/A	N/A	N/A	N/A	N/A	N/A	146 (100)**	N/A
Pelletier, *et al* (2006)	45	31 (26.3)	87 (73.7)	14 (11.8)	104 (88)*	43 (36.4)	75 (63.6)	N/A	3.8 (2.8)
Vanrenterghem, *et at* (2005)	46.3	285 (34.2)	548 (65.8)	N/A	818 (98.2)	65 (7.8)	768 (92.2)	751 (90.2)	2.7 (N/A)
Smak Gregoor, *et al* (2002)	51.6	72 (34)	140 (66)	N/A	N/A	52 (24.5)	160 (75.5)	189 (89.1)	0.8 (0.6)
Sola, *et al* (2002)	N/A	N/A	N/A	N/A	N/A	N/A	92 (100)	N/A	N/A
Boletis, *et al* (2001)	40.1	20 (30.3)	46 (69.7)	N/A	N/A	30 (45.4)	36 (54.5)	N/A	8.3 (3.8)
Ahsan, *et al* (1999)	49.9	93 (45)	173 (65)	48 (18.4)	218 (82)*	114 (42.8)	152 (57.2)	N/A	N/A

HLA: Human leukocyte antigens

SD: Standard deviation

* Non-African Americans was specified in the study

** The study reads de novo renal transplant patients

Study quality

All included trials were published in peer-reviewed publications. One study was published in an abstract form [3]. Two studies were double-blind trials [6, 11]; one study was a single-blind trial [4]; and six studies were open-label RCTs [3, 5, 7-10]. Four studies did not report the source of funding [3, 8-10]; and five of the trials were partially or completely funded by pharmaceutical companies [4-7, 11].

Clinical outcomes

During an average follow-up of 15 (range: 6–24) months after transplantation, a total of 86 acute graft rejections (60 in the corticosteroid withdrawal group, and 26 in the corticosteroid maintenance group); 48 graft failures (20 and 28, respectively); 26 all-cause deaths (12 and 14, respectively); and 34 post-transplant diabetes mellitus (16 and 18, respectively) were observed (Table 3). Compared to maintenance therapy, late corticosteroid withdrawal is associated with statistically non-significant 34% increased risk of acute graft rejection (OR=1.34; 95% CI: 0.47–3.82). Absolute risk reduction (ARR) was 4.25% and the number needed to treat (NNT) was about 24.

**Table 3:** Comparing the two corticosteroid therapy strategies in terms of number needed to treat (NNT).

**Clinical Outcome**	**Corticosteroid Withdrawal Group**			**Corticosteroid Maintenance Group**			**ARR** ^#^	**NNT** **
	**No. of Events**	**No. of Patients**	**AR** *	**No. of Events**	**No. of Patients**	**AR** *		
Acute Graft Rejection	60	705	8.51	26	610	4.26	4.25	23.5
Graft Failure	20	537	3.72	28	589	4.75	1.03	97
All-Cause Mortality	12	561	2.14	14	600	2.33	0.19	526
PTDM	16	345	4.64	18	323	5.57	0.93	107.5

AR: Absolute risk, ARR: Absolute risk reduction

NNT: Number needed to treat, PTDM: Post-transplant diabetes mellitus

* Values are in percentages

# Calculated by subtracting the AR values in the maintenance group from the AR values in the withdrawal group

** Equals 1/ARR%.

Late corticosteroid withdrawal was associated with both statistically non-significant 35% and 5% reductions in the risks of graft failure and all-cause mortality (OR=0.65; 95% CI: 0.26–1.60; OR=0.95; 95% CI: 0.23–3.93, respectively). ARR and NNT for graft failure were 1.03% and 97, and those for all-cause mortality were 0.19% and 526.

Stopping corticosteroids at least three months after kidney transplantation was associated with statistically non-significant 4% increased risk of post-transplant diabetes mellitus compared to the corticosteroid maintenance therapy (OR=1.04; 95% CI: 0.45–2.41). ARR was 0.93%, and NNT was about 108.

A statistically significant reduction in the mean total cholesterol was greater in the corticosteroid withdrawal group (mean difference: 18.1 mg/dL; 95% CI: 7.1–29.0 mg/dL), however; no reduction in serum creatinine was observed (0.00 mg/dL; 95% CI, 0.17 to 0.17 mg/dL) in comparison to the maintenance therapy group.

**Figure 3:**
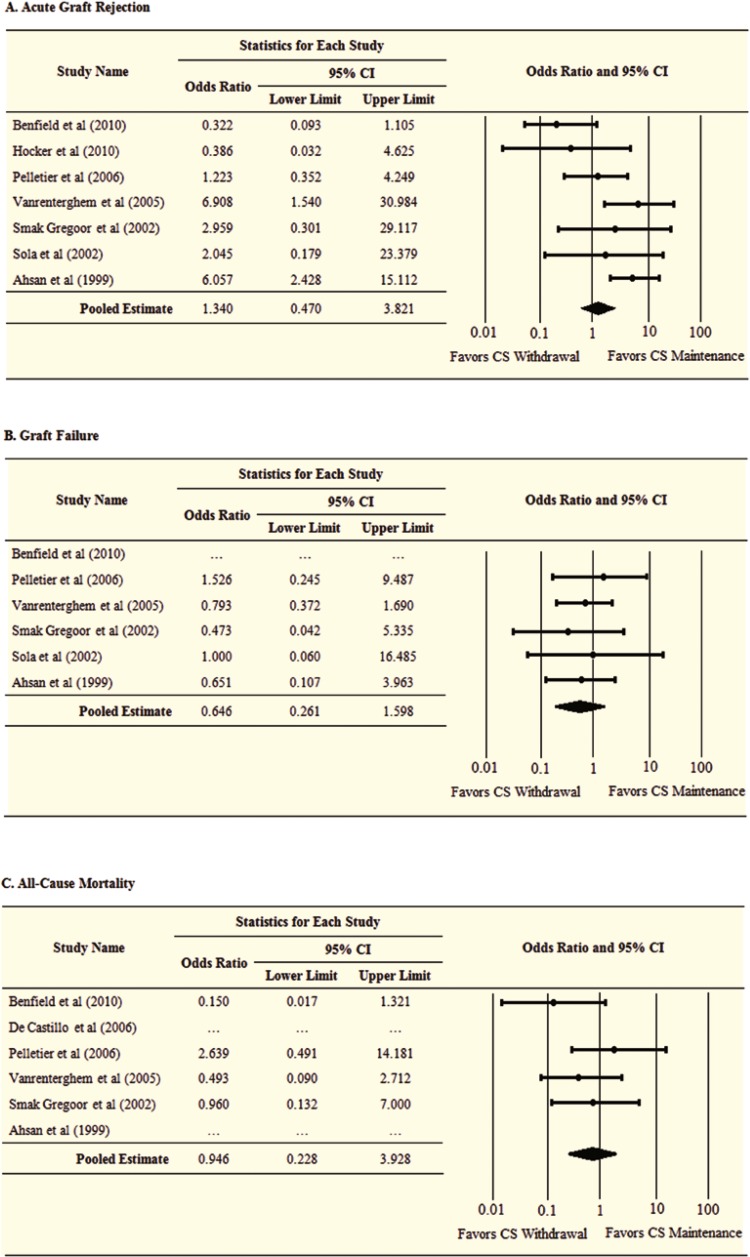
Analysis of clinical outcomes following late corticosteroid withdrawal therapy vs. corticosteroid maintenance therapy in renal transplant patients. Acute graft rejection (A); graft failure (B); and all-cause mortality (C

**Figure 4:**
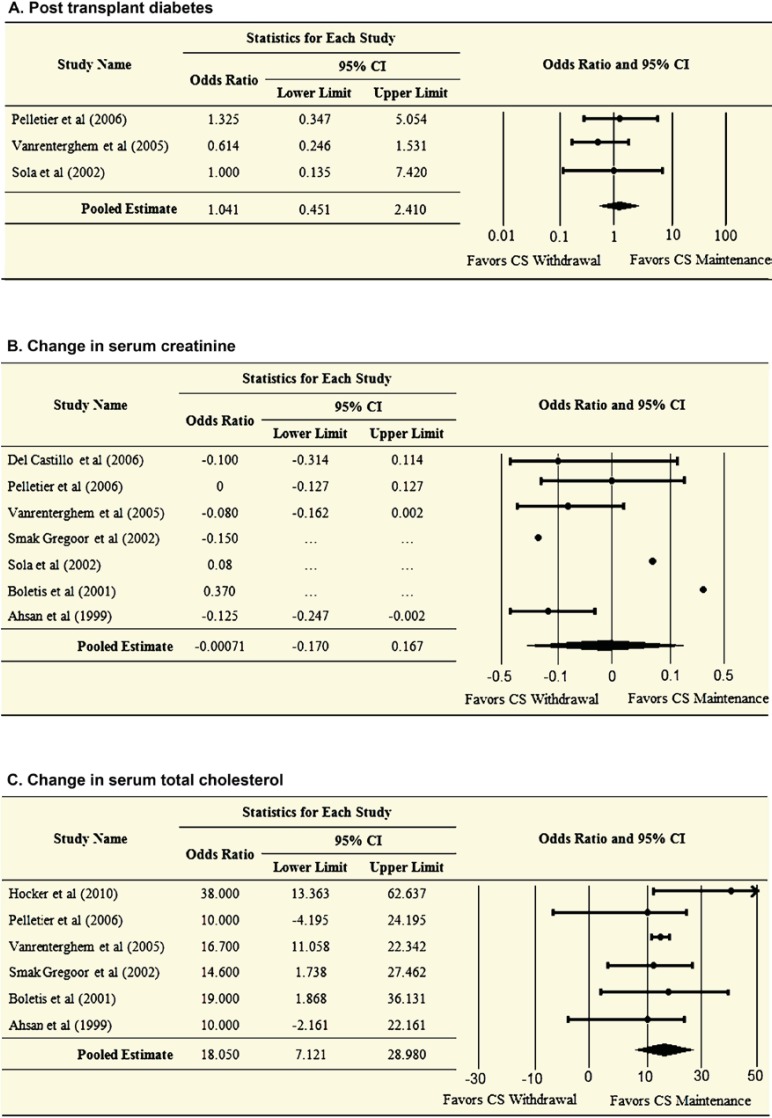
Analysis of clinical outcomes following late corticosteroid withdrawal therapy vs. corticosteroid maintenance therapy in renal transplant patients. Post-transplant diabetes (A); change in serum creatinine (B); and change in total cholesterol (C).

Table 3 compares the two corticosteroid strategies in terms of NNT; Figures 3 and 4 display the pooled meta-analysis estimates for the clinical outcomes of interest. Furthermore, two clinical trials involved pediatric patients, which reported the change in standardized height z-score as a measure for intervention effect on growth [6, 7]. Both studies showed that late corticosteroid withdrawal is associated with better growth outcomes compared to corticosteroid maintenance therapy. We did not pool the estimates of both studies.

## DISCUSSION

It is known that continued immunosuppression therapy with corticosteroids in kidney transplant patients reduces the risk of acute graft rejection, however; the long-term complications of such therapy are well-established [12]. Prolonged corticosteroid therapy is associated with a spectrum of adverse outcomes, including opportunistic infections, hyperglycemia, hyperlipidemia, hypertension, glaucoma, cataracts, hypercoagulability of the blood, muscle wasting and weakness, osteoporosis, Cushing’s syndrome, and growth retardation in children. It is hypothesized that minimizing immunosuppression by gradually withdrawing corticosteroids contributes to a reduction in long-term risks [14]. We conducted a meta-analysis of published RCTs of kidney transplant patients, who were randomized to late corticosteroid withdrawal (at >3 months post-transplantation) therapy or corticosteroid maintenance therapy under continuation of concurrent immunosuppression by either TAC or MMF, or both. The clinical outcomes of interest included the incidence of acute graft rejection; graft failure; all-cause mortality; post-transplant diabetes; changes in serum creatinine and total cholesterol levels; and changes in standardized z-score of height in pediatric patients.

Although statistically not significant, our findings indicate that late corticosteroid withdrawal in kidney transplant patients is associated with 34% increase in the risk of acute graft rejection (95% CI for OR: 0.47–3.82); and 4% increase in the risk of post-transplant diabetes mellitus (95% CI for OR: 0.45–2.41). However, this strategy resulted in 35% and 5% reductions in the risk of graft failure and patient’s all-cause mortality, respectively (95% CI for OR: 0.26–1.60; and 95% CI for OR: 0.23–3.93, respectively). Stopping corticosteroid therapy was associated with a statistically significant substantial reduction in total cholesterol levels (mean difference: 18.05 mg/dL; 95% CI: 7.12–29.0 mg/dL), but did not reduce serum creatinine levels (0.00 mg/dL; 95% CI: 0.17 to 0.17). Two studies showed that corticosteroids withdrawal was presented with better growth outcomes in pediatric kidney transplant patients [6, 7].

Moreover, late corticosteroid withdrawal can be compared with maintenance therapy in terms of more clinically significant measure, the NNT (Table 3), which can be interpreted as the average number of patients that is required to withdraw corticosteroid therapy at least three months after kidney transplantation and followed for an average of 15 months to observe one less clinical outcome of interest by the end of the 15-month period. To illustrate, for acute graft rejection, the kidney transplant specialist would need 24 kidney transplant patients, on average, to withdraw corticosteroid in order to prevent one acute rejection of the transplanted kidney. The same applies for graft failure, all-cause mortality, and post-transplant incidence of diabetes mellitus; on average, 97 patients are needed to stop corticosteroid treatment to prevent the occurrence of one case of graft failure; 526 patients are needed to withdraw corticosteroid treatment to observe one less death event; and about 108 patients are required to stop corticosteroid therapy in order to prevent one case of new-onset diabetes mellitus at the end of the follow-up time. It is clear that larger NNT values are indicators of less effective interventions. In another word, it requires several patients to be exposed to the intervention (corticosteroid withdrawal) to observe one additional success, on average, compared to the standard alternative (corticosteroid maintenance).

Our study demonstrates statistically non-significant results that show late corticosteroid withdrawal could reduce the risks of long-term complications associated with immunosuppression in kidney transplant patients, however; it did not show that such strategy could reduce the risk of acute graft rejections and the incidence of diabetes mellitus after kidney transplantation. Nevertheless, it shows clinically significant end-points in terms of NNT values for acute rejection and graft failure.

The present meta-analysis has several limitations. First, the results should be interpreted with caution, because many variables were not uniformly distributed across the selected trials. Heterogeneity was apparent in terms of study population characteristics, follow-up and randomization times, outcome measurement time, and treatment regimen characteristics. These differences imparted to the statistical differences in the effect size between the individual studies (Figures 3 and 4). Second, our analysis did not have sufficient statistical power to detect statistically significant differences between the late corticosteroid withdrawal group and the corticosteroid maintenance group. This was mainly attributed to the fact that included trials did not have large number of enrollees. Third, we did not include unpublished evidence, which might have more recent findings, however, unpublished literatures more likely have statistically non-significant findings, which prevent them from reaching the information database, *i.e.*, publication bias. Unlike published studies, unpublished works could impart bias because they are not subjected to the peer review process. Finally, meta-analysis is a retrospective research that is highly dependent upon the inherent limitations, strengths, and quality of the included studies. A detailed study protocol was developed prior to the literature search, data extraction, and data analysis phases, which minimized the potential for systematic errors.

Given the heterogeneity between the trials, a random effects model was deemed appropriate. In addition, unweighted method is considered more valid in meta-analysis research, because the weights are random entities, and should not be treated as fixed alternatives, which unfortunately is the case in most of the published meta-analyses.

## CONCLUSIONS

Late corticosteroid withdrawal under TAC and/or MMF-lead immunosuppression after kidney transplantation could provide benefits in terms of total cholesterol, patient and graft survival, and pediatric growth. This strategy, however did not reduce the risk of acute graft rejection, post-transplant diabetes mellitus, and deterioration in serum creatinine levels. Additional large scale, multicenter RCTs are required to further evaluate the long-term clinical outcomes associated with late corticosteroid withdrawal and assessing corticosteroid-sparing effects of TAC and MMF in kidney transplant patients.
